# Systematic review of diagnostic and prognostic host blood transcriptomic signatures of tuberculosis disease in people living with HIV

**DOI:** 10.12688/gatesopenres.14327.2

**Published:** 2023-05-05

**Authors:** Simon C Mendelsohn, Savannah Verhage, Humphrey Mulenga, Thomas J Scriba, Mark Hatherill

**Affiliations:** 1 South African Tuberculosis Vaccine Initiative, Institute of Infectious Disease and Molecular Medicine and Division of Immunology, Department of Pathology, University of Cape Town, Cape Town, Western Cape, 7935, South Africa

**Keywords:** Host, blood, diagnostic, prognostic, gene, signatures, tuberculosis, HIV

## Abstract

Background

HIV-associated tuberculosis (TB) has high mortality; however, current triage and prognostic tools offer poor sensitivity and specificity, respectively. We conducted a systematic review of diagnostic and prognostic host-blood transcriptomic signatures of TB in people living with HIV (PLHIV).

Methods

We systematically searched online databases for studies published in English between 1990-2020. Eligible studies included PLHIV of any age in test or validation cohorts, and used microbiological or composite reference standards for TB diagnosis. Inclusion was not restricted by setting or participant age. Study selection, quality appraisal using the QUADAS-2 tool, and data extraction were conducted independently by two reviewers. Thereafter, narrative synthesis of included studies, and comparison of signatures performance, was performed.

Results

We screened 1,580 records and included 12 studies evaluating 31 host-blood transcriptomic signatures in 10 test or validation cohorts of PLHIV that differentiated individuals with TB from those with HIV alone, latent *Mycobacterium tuberculosis* infection, or other diseases (OD). Two (2/10; 20%) cohorts were prospective (29 TB cases; 51 OD) and 8 (80%) case-control (353 TB cases; 606 controls) design. All cohorts (10/10) were recruited in Sub-Saharan Africa and 9/10 (90%) had a high risk of bias. Ten signatures (10/31; 32%) met minimum WHO Target Product Profile (TPP) criteria for TB triage tests. Only one study (1/12; 8%) evaluated prognostic performance of a transcriptomic signature for progression to TB in PLHIV, which did not meet the minimum WHO prognostic TPP.

Conclusions

Generalisability of reported findings is limited by few studies enrolling PLHIV, limited geographical diversity, and predominantly case-control design, which also introduces spectrum bias. New prospective cohort studies are needed that include PLHIV and are conducted in diverse settings. Further research exploring the effect of HIV clinical, virological, and immunological factors on diagnostic performance is necessary for development and implementation of TB transcriptomic signatures in PLHIV.

## Introduction

There were an estimated 703,000 HIV-associated incident tuberculosis (TB) cases in 2021 however only 368,600 (52%) were notified, with a resultant case fatality rate of 27% ^1^. Earlier diagnosis and initiation of treatment, or disease prevention through targeted short-course TB preventive therapy (TPT), may reduce this burden. However, we lack adequate TB mass screening tools to direct confirmatory testing or prognostic tools to guide preventive therapy in the outpatient or community setting. Symptom screening, the most widely used TB triage tool, has low specificity in antiretroviral therapy (ART-) naïve and low sensitivity in ART-experienced people living with HIV (PLHIV) ^2^. The addition of chest radiography improves sensitivity, at a cost of reduced specificity ^2^. With almost three-quarters of the 38 million PLHIV globally now receiving ART ^3^, new tools should be efficacious in this group.

The WHO currently recommends that PLHIV with a positive or unknown tuberculin skin test (TST) result should receive TPT, if active TB has been excluded ^4^. There is strong evidence to support such an approach ^5^. However, TST and interferon-γ release assay (IGRA) reflect a memory T-cell response following *Mycobacterium tuberculosis* (Mtb) exposure (sensitisation) and not necessarily ongoing infection. In TB-endemic countries with high rates of Mtb transmission and exposure, these tests have limited utility for guiding TPT ^6, 7^. In addition, loss or dysfunction of Mtb-specific memory T-cells among immunocompromised PLHIV with low CD4 cell counts results in lower IGRA positivity and may reduce sensitivity for predicting progression to disease ^8, 9^. There is also limited evidence regarding repeat courses of TPT among PLHIV; a recent study demonstrated that universal retreatment after one year did not provide additional benefit ^10^.

Biomarker-guided treatment has been proposed to target therapy to those that need it most, reducing unnecessary pill burden, drug interactions, and side effects in individuals, and increasing efficacy and cost-effectiveness of mass screening ^11, 12^. Host-response blood transcriptomic signatures can identify those with active TB and those who are progressing to disease ^13– 15^. Performance of most signatures in adults without HIV meet at least one of the minimum World Health Organization (WHO) Target Product Profile (TPP) TB triage test performance criteria (sensitivity 90% and specificity 70%) for diagnosing prevalent TB ^16, 17^. Several signatures have been shown to meet minimum prognostic benchmarks (sensitivity 75% and specificity 75%) ^18^ for short-term prediction of progression to TB disease within six months of testing ^19– 21^. However, only a couple of signatures, Roe1 and Roe3 ^22, 23^, meet these criteria through 12 months of follow-up for progression ^21^. Transcriptomic biomarkers selected for advancement through the diagnostics pipeline for development as point-of-care assays should also perform well in PLHIV. We systematically reviewed the published literature on host-response blood transcriptomic biomarkers for diagnosing prevalent and predicting progression to incident TB disease in PLHIV, and compared performance to the WHO TPP criteria.

## Methods

### Protocol and registration

This review is reported in line with the Preferred Reporting Items for Systematic reviews and Meta-Analysis of Diagnostic Test Accuracy Studies (PRISMA-DTA) ^24^ recommendations ( Table 1). The systematic review protocol was registered with the International Prospective Register of Systematic Reviews (PROSPERO) on 02 January 2021 with registration number CRD42021224155 and published in the *BMJ Open* ^25^.

**Table 1.** PRISMA for Diagnostic Test Accuracy 2018 checklist. **This checklist has been adapted from** McInnes MDF, Moher D, Thombs BD, McGrath TA, Bossuyt PM, The PRISMA-DTA Group (2018). Preferred Reporting Items for a Systematic Review and Meta-analysis of Diagnostic Test Accuracy Studies: The PRISMA-DTA Statement. JAMA. 2018 Jan 23;319(4):388–396. doi: 10.1001/jama.2017.19163.

Section/topic	#	PRISMA-DTA Checklist Item	Reported on page #
**TITLE / ABSTRACT**			
Title	1	Identify the report as a systematic review (+/- meta-analysis) of diagnostic test accuracy (DTA) studies.	Page 1
Abstract	2	Abstract: See PRISMA-DTA for abstracts.	Page 1
**INTRODUCTION**			
Rationale	3	Describe the rationale for the review in the context of what is already known.	Page 3
Clinical role of index test	D1	State the scientific and clinical background, including the intended use and clinical role of the index test, and if applicable, the rationale for minimally acceptable test accuracy (or minimum difference in accuracy for comparative design).	Page 3
Objectives	4	Provide an explicit statement of question(s) being addressed in terms of participants, index test(s), and target condition(s).	Page 3
**METHODS**			
Protocol and registration	5	Indicate if a review protocol exists, if and where it can be accessed (e.g., Web address), and, if available, provide registration information including registration number.	Page 3
Eligibility criteria	6	Specify study characteristics (participants, setting, index test(s), reference standard(s), target condition(s), and study design) and report characteristics (e.g., years considered, language, publication status) used as criteria for eligibility, giving rationale.	Page 3
Information sources	7	Describe all information sources (e.g., databases with dates of coverage, contact with study authors to identify additional studies) in the search and date last searched.	Page 3
Search	8	Present full search strategies for all electronic databases and other sources searched, including any limits used, such that they could be repeated.	Page 3
Study selection	9	State process for selecting studies (i.e. screening, eligibility, included in systematic review, and, if applicable, included in meta-analysis).	Page 4
Data collection process	10	Describe method of data extraction from reports (e.g., piloted forms, independently, in duplicate) and any processes for obtaining and confirming data from investigators.	Page 4
Definitions for data extraction	11	Provide definitions used in data extraction and classifications of target condition(s), index test(s), reference standard(s) and other characteristics (e.g. study design, clinical setting).	Page 3
Risk of bias & applicability	12	Describe methods used for assessing risk of bias in individual studies and concerns regarding the applicability to the review question.	Page 6
Diagnostic accuracy measures	13	State the principal diagnostic accuracy measure(s) reported (e.g. sensitivity, specificity) and state the unit of assessment (e.g. per-patient, per-lesion).	Page 5
Synthesis of results	14	Describe methods of handling data, combining results of studies and describing variability between studies. This could include, but is not limited to: a) handling of multiple definitions of target condition. b) handling of multiple thresholds of test positivity, c) handling multiple index test readers, d) handling of indeterminate test results, e) grouping and comparing tests, f) handling of different reference standards	Page 5
Meta-analysis	D2	Report the statistical methods used for meta-analyses, if performed.	NA
Additional analyses	16	Describe methods of additional analyses (e.g., sensitivity or subgroup analyses, meta- regression), if done, indicating if pre-specified.	NA
**RESULTS**			
Study selection	17	Provide numbers of studies screened, assessed for eligibility, included in the review (and included in meta-analysis, if applicable) with reasons for exclusions at each stage, ideally with a flow diagram.	Page 6 Figure 1
Study characteristics	18	For each included study provide citations and present key characteristics including: a) participant characteristics (presentation, prior testing), b) clinical setting, c) study design, d) target condition definition, e) index test, f) reference standard, g) sample size, h) funding sources	Table 2, Table 3
Risk of bias and applicability	19	Present evaluation of risk of bias and concerns regarding applicability for each study.	Page 14, Figure 2
Results of individual studies	20	For each analysis in each study (e.g. unique combination of index test, reference standard, and positivity threshold) report 2x2 data (TP, FP, FN, TN) with estimates of diagnostic accuracy and confidence intervals, ideally with a forest or receiver operator characteristic (ROC) plot.	Figure 3– Figure 6
Synthesis of results	21	Describe test accuracy, including variability; if meta-analysis was done, include results and confidence intervals.	Pages 14–17
Additional analysis	23	Give results of additional analyses, if done (e.g., sensitivity or subgroup analyses, meta-regression; analysis of index test: failure rates, proportion of inconclusive results, adverse events).	NA
**DISCUSSION**			
Summary of evidence	24	Summarize the main findings including the strength of evidence.	Pages 17–19
Limitations	25	Discuss limitations from included studies (e.g. risk of bias and concerns regarding applicability) and from the review process (e.g. incomplete retrieval of identified research).	Pages 17–19
Conclusions	26	Provide a general interpretation of the results in the context of other evidence. Discuss implications for future research and clinical practice (e.g. the intended use and clinical role of the index test).	Pages 17–19
**FUNDING**			
Funding	27	For the systematic review, describe the sources of funding and other support and the role of the funders.	Page 2

### Eligibility criteria

We considered cross-sectional and case-control studies, prospective and retrospective cohort studies, and randomised control trials evaluating diagnostic and/or prognostic performance of human host-blood transcriptomic signatures of TB (index tests). Eligible studies included PLHIV in the signature test and/or validation cohorts. Studies that only reported signature discovery cohort performance, or treatment response and failure monitoring cohorts, were not considered. PLHIV of all ages, ethnicities, and in all settings were considered. Studies which did not report any measures of signature performance (sensitivity and specificity, or reported data which enable the reconstruction of a two-by-two table for test accuracy calculation for PLHIV), did not clearly state the case definition of TB disease, did not report primary data, or did not independently report signature performance in PLHIV, were excluded.

### Endpoint definitions

The primary TB disease endpoint (target condition) was defined by a positive microbiological test, such as mycobacterial culture or the Xpert MTB/RIF assay (reference standards), in sputum or other bodily fluid sample. Microbiologically-confirmed extra-pulmonary TB disease was also considered. The secondary TB disease endpoint was defined by non-microbiologically-confirmed, presumptive TB diagnosed via composite clinical features. TB disease diagnosed within one month of the index test was presumed to be prevalent disease (diagnostic studies). Prognostic studies were defined as prospective studies in which participants were followed up for progression to incident TB disease with measurement of a transcriptomic signature from blood samples collected at enrolment. Eligible studies included healthy individuals, latent Mtb-infected individuals, or individuals with other respiratory or systemic diseases as a control group. Latent Mtb infection was defined by a positive TST or IGRA.

### Search strategy and information sources

We systematically searched PubMed ( *MEDLINE*), *WOS Core Collection, Biological Abstracts,* and *SciELO Citation Index (via Web of Science), Africa-Wide Information* and *General Science Abstracts (via EBSCOhost)*, *Scopus*, and *Cochrane Central Register of Controlled Trials* databases for full-text articles published in English between 1 January 1990 and 31 December 2020 using Medical Subject Headings (MeSH) and keyword search terms for "Diagnosis", "Messenger RNA", "Biomarkers/blood”, “Tuberculosis”, and “HIV”. The search strategy, including publication date range, were prespecified and published in a systematic review protocol ^25^. We reviewed reference lists of eligible articles and performed forward citation tracking using *Science Citation Index* (via *Web of Science*) to identify further articles and reports missed by the electronic database search ^26^.

### Study selection and data collection

Two reviewers (SCM and SV) independently conducted the literature search and screened the search outputs for potential inclusion using EndNote bibliographic software to manage references, as previously described ^27^. After removal of duplicates, the selection process included an initial screening of titles and abstracts for relevance, followed by full text review for eligibility. The two reviewers resolved any disagreements or uncertainties by discussion. Data elements of included studies were then independently extracted by the two reviewers. Corresponding authors of potentially eligible studies were contacted to provide deidentified participant-level data to reconstruct two-by-two tables or summary performance data for the PLHIV subgroup. Studies without summary or participant-level data available for the PLHIV subgroup were excluded.

### Data analysis

We performed a narrative synthesis of the eligible study cohorts and signatures, including study design, cohort and signature characteristics, and diagnostic and prognostic performance of signatures stratified by study control groups (healthy, latent-Mtb infected, or other disease), and diagnostic reference standards (microbiological or composite clinical). Studies and cohorts were designated by the first author name and year of publication (e.g. Author2019a) and signatures by first author and number of transcripts (e.g. Author11). Signature area under the curve (AUC), sensitivity, and specificity were summarised in forest plots (R *forestplot* package ^28^). For studies with available participant-level data for the PLHIV subgroup, we were able to recalculate AUC (R *pROC* package ^29^), and benchmark sensitivity and specificity against the WHO TPP minimum performance criteria for a triage (70% specificity and 90% sensitivity) ^16^ or prognostic (75% specificity and 75% sensitivity) ^18^ test. 95% Confidence intervals for AUCs and sensitivity and specificity, were calculated using the DeLong ^30^ and Wilson binomial ^31^ methods, respectively. For studies in which participant-level data were not available, we report summary AUC, sensitivity, and specificity estimates, and 95% confidence intervals, for the PLHIV subgroup as published in the original papers. Most of these estimates were not specifically benchmarked against the WHO TPP minimum performance criteria for a triage test.

### Risk of bias, applicability, and quality of evidence

The methodological quality and applicability concerns of included studies was assessed by the two reviewers using the Quality Assessment of Diagnostic Accuracy Studies-2 (QUADAS-2) tool ^32^ and graphically represented using traffic-light plots (R *robvis* package ^33^). Risk of bias and applicability concerns for individual study was evaluated in four domains relating to (1) patient selection, (2) measurement of the index test, (3) measurement of the reference standard, and (4) study flow and timing of investigational and diagnostic procedures. Risks of bias are reported within each domain of each study as low risk (no risks of bias identified), some concerns (one risk or unclear risk of bias identified), or high risk (more than one risk or unclear risk of bias identified). Overall risk of bias for each study was reported as low risk (no risks of bias identified in any domains), some concerns (some concerns identified in one domain), or high risk (some concerns identified in two or more domains or high risk of bias in any domain). We assessed the cumulative quality of evidence synthesised by the systematic review using the “Grading of Recommendations Assessment, Development and Evaluation” (GRADE) approach ^34^ with classification based on study design and limitations, indirectness, inconsistency, imprecision, and publication bias ^35, 36^.

## Results

### Search results

**Figure 1.**
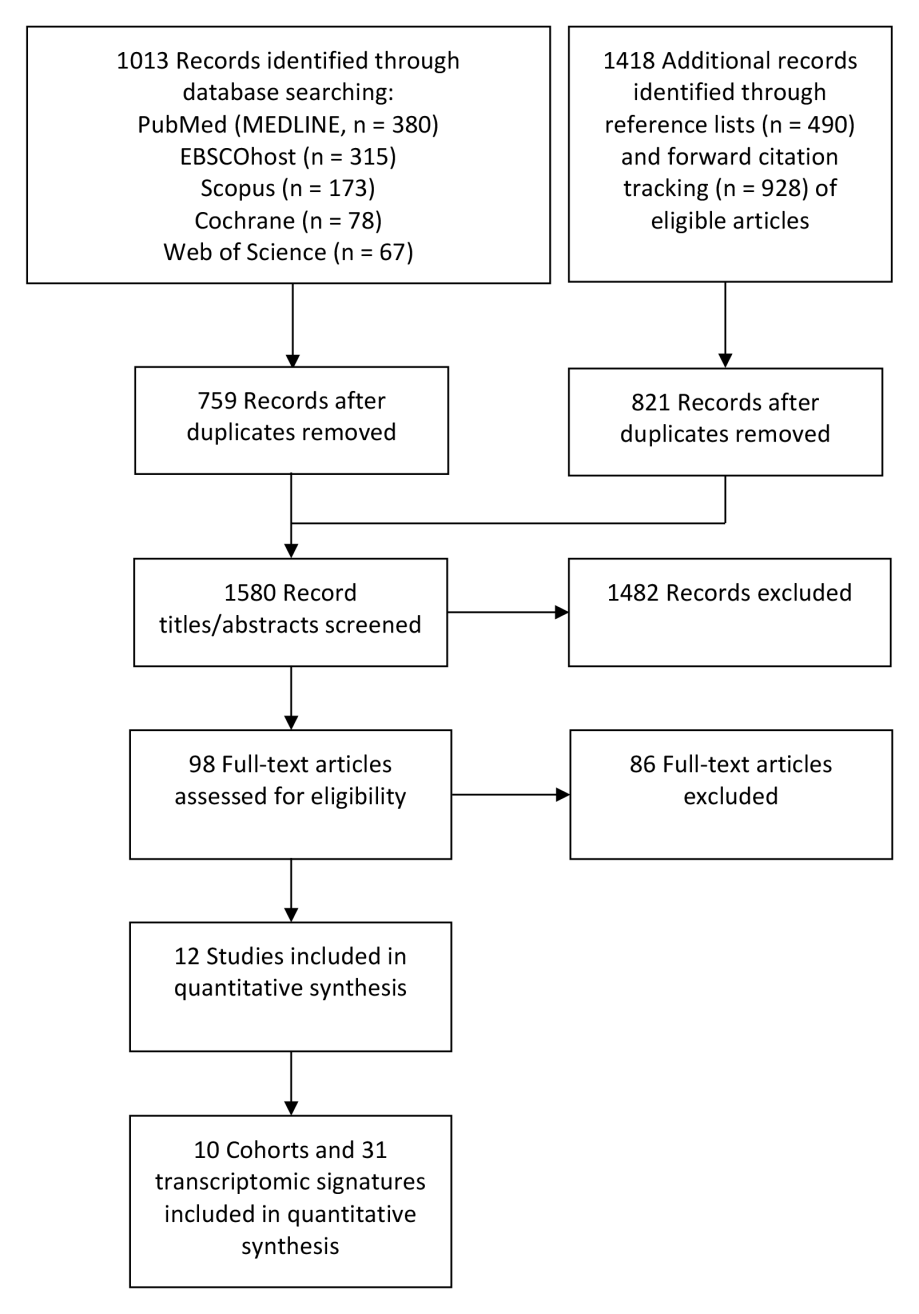
Preferred Reporting Items for Systematic Reviews and Meta-Analyses (PRISMA) flow diagram.

We performed the literature search in January 2021, identifying 1,580 unique records published between 1 January 1990 and 31 December 2020, of which 98 full-text articles were assessed for eligibility, and 12 studies ^13, 22, 37– 47^ met all criteria for inclusion ( Figure 1). The main reasons for study exclusion were absence of PLHIV in study cohorts (n=29), absence of an independent test or external validation cohort which included PLHIV (n=20), and inappropriate index test (n=16) or study design (n=10). Nine of 10 studies excluded for inappropriate design were commentaries or reviews. In addition, deidentified participant-level data to reconstruct two-by-two tables, or summary performance data for PLHIV, were not available and no data were received from corresponding authors for 9 records ^19, 48– 55^.

### Study cohorts included in quantitative synthesis

The 12 eligible studies included 10 independent test or validation cohorts featuring PLHIV ( Table 2), cumulatively evaluating diagnostic performance of 31 transcriptomic signatures ( Table 3) incorporating over 700 unique transcripts. All independent test and validation cohorts enrolled PLHIV from outpatient clinics or hospital inpatients, most with suspicion (symptom-positive) or high risk (initiating antiretroviral therapy) of prevalent TB ( Table 2). No studies prospectively enrolled participants without significant risk factors from community settings with a high burden of subclinical TB. Cohort sizes were generally small, with a maximum of 97 TB cases and 176 controls with HIV in the Kaforou2013 ^37^ study, and 67 TB cases and 134 controls with HIV in the Södersten2020 ^46^ study. All other cohorts included less than 50 TB cases with HIV. Only one of the 12 studies (Darboe2019) ^42^ reported transcriptomic signature prognostic performance for incident TB disease (disease recurrence after completion of TB treatment). The remainder reported diagnostic performance for differentiating patients with pulmonary TB disease from healthy controls, from controls with latent TB infection, or from other diseases. Most studies were conducted in Africa, with 8 out of 10 cohorts enrolling participants in South Africa. Only one cohort (Södersten2020) ^46^ included participants from another continent (Peru, South America). Many studies used the same microarray datasets, GSE37250 ^37^ and GSE39941 ^38^, from the Kaforou2013 (Malawi and South Africa) and Anderson2014 (Kenya, Malawi and South Africa) studies respectively, for external validation of signatures. Three cohorts measured signature scores using near-point-of-care RT-qPCR platforms (Darboe2019 and Penn-Nicholson2019a,b) ^42, 45^, while only one study (Södersten2020) ^46^ used a point-of-care device, the Cepheid Xpert-MTB Host-Response (HR) Prototype, to measure the Sweeney3 signature ^19, 49^ in biobanked whole blood RNA samples. No included studies validated signature performance for diagnosing extrapulmonary TB. However, one cohort (Anderson2014b) ^38^ included 17 HIV-infected children with non-microbiologically-confirmed, presumptive clinical TB with clinical and radiologic features that prompted empirical treatment. All other study cohorts, with the exception of Anderson2014, enrolled adults and used a microbiological reference standard (culture or Xpert MTB/RIF).

**Table 2.** Study cohorts included in quantitative synthesis.

Cohort ID *	Cohort name or accession number	Study design	Study setting	Country	Age group	Cohort type	Cases	Controls	Sample type	Signature measurement method	HIV+ TB cases	HIV+ controls
**Anderson2014a ^38^**	GSE39941	Case-control nested within prospective cohort	Children with suspected TB (Hospital setting)	Malawi, South Africa	Children	Diagnostic	Sputum culture+	OD	Whole blood	Microarray	40	65
**Anderson2014b ^38^**	GSE39941	Case-control nested within prospective cohort	Children with suspected TB (Hospital setting)	Kenya	Children	Diagnostic	Sputum culture+ (n=10) or composite reference standard (n=17)	OD	Whole blood	Microarray	27	27
**Darboe2019 ^42^**	TRuTH Cohort	Case-control nested within prospective cohort	Outpatients who recently completed TB treatment	South Africa	Adults	Prognostic	Sputum culture+ recurrent TB within 3 years of treatment completion	No recurrent TB	PBMC	RT-qPCR	38	84
**Kaforou2013a ^37^**	GSE37250	Case-control study	Hospital inpatient, community, clinic	Malawi, South Africa	Adults	Diagnostic	Sputum culture+	LTBI	Whole blood	Microarray	97	84
**Kaforou2013b ^37^**	GSE37250	Case-control study	Hospital inpatient, community, clinic	Malawi, South Africa	Adults	Diagnostic	Sputum culture+	OD	Whole blood	Microarray		92
**Penn-** **Nicholson2019a ^45^**	CTBC Cohort	Case-control study	Clinic outpatient	South Africa	Adults	Diagnostic	Sputum culture+ or Xpert MTB/RIF+	LTBI+HC	Whole blood	RT-qPCR	44	40
**Penn-** **Nicholson2019b ^45^**	ScreenTB and AE-TBC Cohorts	Prospective cohort	Symptomatic clinic attendees	South Africa	Adults	Diagnostic	Sputum culture+ or Xpert MTB/RIF+	ORD	Whole blood	RT-qPCR	12	24
**Rajan2018 ^44, 47^**	Data not publicly available	Case-control nested within prospective cohort	Outpatients initiating ART	Uganda	Adults	Diagnostic	Sputum culture+	LTBI+HC	Whole blood	Microarray	40	80
**Södersten2020 ^46^**	Biobanked PAXgene samples	Nested case- control study	Symptomatic inpatients	Peru, South Africa	Adults	Diagnostic	Sputum/blood culture+ or sputum/urine Xpert MTB/RIF+	OD	Whole blood	Cepheid Xpert-MTB-HR- Prototype	67	134
**Turner2020 ^13^**	Array Express: E-MTAB-8290	Prospective cohort	Symptomatic clinic attendees	South Africa	Adults	Diagnostic	Sputum culture+ or Xpert MTB/RIF+	ORD	Whole blood	RNA sequencing	17	27

* A study may evaluate multiple signatures using several validation cohorts. Studies and cohorts are designated by the first author name and year of publication (e.g. Author2019a) and signatures by first author and number of transcripts (e.g. Author11). GSE, Genomic Spatial Event (database). TruTH, TB Recurrence upon Treatment with HAART. CTBC, Cross-sectional TB Cohort. AE-TBC, African-European Tuberculosis Consortium. LTBI, latent tuberculosis infection. HC, healthy controls. OD, other diseases. ORD, other respiratory diseases. PBMC, peripheral blood mononuclear cell. RT-qPCR, real-time quantitative polymerase chain reaction.

**Table 3.** Transcriptomic signatures included in quantitative synthesis.

Signature ID *	Year published	Intended use	Signature model	Discovery cohort study design	Discovery cohort country	Discovery cohort age group	Discovery cohort HIV status	Discovery data	Discovery approach
**Anderson42 (39.** **LTBI) ^38^**	2014	Diagnostic (TB vs LTBI)	Disease risk score	Case-control nested in prospective cohort	Malawi, South Africa	Children	Mixed	Genome-wide RNA Microarray	Elastic net
**Anderson51 (39.** **OD) ^38^**	2014	Diagnostic (TB vs OD)	Disease risk score	Case-control nested in prospective cohort	Malawi, South Africa	Children	Mixed	Genome-wide RNA Microarray	Elastic net
**Berry86 ^58^**	2010	Diagnostic (TB vs OD)	k-nearest neighbours algorithm	Case-control	United Kingdom	Adults	Negative	Genome-wide RNA Microarray	Differential expression
**Berry393 ^58^**	2010	Diagnostic (TB vs LTBI+HC)	k-nearest neighbours algorithm	Case-control	South Africa, United Kingdom	Adults	Negative	Genome-wide RNA Microarray	Differential expression
**Darboe11 ^56^**	2018	Prognostic (Incipient TB vs HC)	Pair-wise SVM ensemble	Case-control nested in prognostic cohort	South Africa	Adolescents	Negative	RT-qPCR using selected target gene set (Zak16)	Refinement of Zak16: most reproducible and least redundant genes
**de Araujo1** ** (NPC2) ^59^**	2016	Diagnostic (TB vs HC + LTBI)	Standardised expression	Case-control	Brazil	Adults	Not stated	Genome-wide RNA Sequencing	Differential expression
**Duffy10 ^43^**	2019	Diagnostic (TB vs LTBI + OD)	Six-class multinomial random forest	Case-control	Malawi, South Africa	Adults	Mixed	Genome-wide RNA Microarray	Multinomial random forest
**Gjøen8 (7) ^51^**	2017	Diagnostic (TB vs HC)	LASSO regression	Case-control	India	Children	Negative	198 selected target genes measured by dc-RT MLPA	LASSO
**Gliddon3 ^55^**	2020	Diagnostic (TB vs LTBI)	Disease risk score	Case-control	Malawi, South Africa	Adults	Mixed	Genome-wide RNA Microarray	FS-PLS
**Gliddon4 ^55^**	2020	Diagnostic (TB vs OD)	Disease risk score	Case-control	Malawi, South Africa	Adults	Mixed	Genome-wide RNA Microarray	FS-PLS
**Huang13 (11) ^60^**	2015	Diagnostic (TB vs HC + OD)	SVM	Case-control	United Kingdom	Adults	Negative	Genome-wide RNA Microarray	LASSO, L _1/2_, and elastic net
**Kaforou27 (25) ^37^**	2013	Diagnostic (TB vs LTBI)	Disease risk score	Case-control	Malawi, South Africa	Adults	Mixed	Genome-wide RNA Microarray	Elastic net
**Kaforou44 (39) ^37^**	2013	Diagnostic (TB vs OD)	Disease risk score	Case-control	Malawi, South Africa	Adults	Mixed	Genome-wide RNA Microarray	Elastic net
**Kaforou53 (45) ^37^**	2013	Diagnostic (TB vs OD+LTBI)	Disease risk score	Case-control	Malawi, South Africa	Adults	Mixed	Genome-wide RNA Microarray	Elastic net
**Maertzdorf4 ^48^**	2015	Diagnostic (TB vs HC + LTBI)	Random forest	Case-control	India	Adults	Negative	360 selected target genes measured by RT-qPCR	Random forest
**Penn-** **Nicholson6 ^45^**	2020	Prognostic (Incipient TB vs HC)	Pair-wise ensemble structure	Case-control nested in prognostic cohort	South Africa	Adolescents	Negative	Genome-wide RNA Sequencing and targeted RT-qPCR	SVM-based gene pair ratios approach
**Qian17 ^50^**	2016	Diagnostic (TB vs HC + OD)	Sum of standardised expression	Case-control	United Kingdom	Adults	Negative	862 Nrf2-mediated genes measured by RNA Microarray	Differential expression
**Rajan5 ^44^**	2018	Diagnostic (HIV+TB vs HIV+HC)	Unsigned sums	Case-control nested in prospective cohort	Uganda	Adults	Positive	Genome-wide RNA Microarray	Differential expression
**Roe1 (BATF2) ^22^**	2016	Diagnostic (TB vs HC and TB patients 2-4 years post recovery)	Standardised expression	Case-control	United Kingdom	Adults	Negative	Genome-wide RNA Microarray	SVM (linear kernel)
**Roe3 ^23^**	2020	Prognostic (Incipient TB vs HC)	SVM (linear kernel)	Case-control	United Kingdom	Adults	Negative	Genome-wide RNA Sequencing	Stability selection and SVM
**Roe4 ^22^**	2016	Diagnostic (TB vs OD)	SVM (linear kernel)	Case-control	United Kingdom	Adults	Negative	Genome-wide RNA Microarray	SVM (linear kernel)
**Roe5 ^22^**	2016	Diagnostic (TB vs HC + OD)	SVM (linear kernel)	Case-control	United Kingdom	Adults	Negative	Genome-wide RNA Microarray	SVM (linear kernel)
**Sambarey10 ^41^**	2017	Diagnostic (TB vs HC + LTBI)	Linear discriminant analysis	Case-control	India	Adults	Negative	Genome-wide RNA Sequencing	Human Protein– Protein Interaction Network, Condition- specific Weighted Networks, Differential expression
**Singhania20 ^53^**	2018	Diagnostic (TB vs LTBI)	Disease risk score (modified)	Case-control	United Kingdom	Adults	Negative	Genome-wide RNA Sequencing	Random forest using modular approach
**Suliman2 ^61^**	2018	Prognostic (Incipient TB vs HC)	Pair-wise ensemble structure	Case-control nested in prognostic cohort	The Gambia, South Africa	Adults	Negative	Genome-wide RNA Sequencing	SVM-based gene pair ratios approach
**Suliman4 ^61^**	2018	Prognostic (Incipient TB vs HC)	Pair-wise ensemble structure	Case-control nested in prognostic cohort	The Gambia, Ethiopia, South Africa	Adults	Negative	Genome-wide RNA Sequencing	SVM-based gene pair ratios approach
**Sweeney3 ^19, 49^**	2016	Diagnostic (TB vs HC + LTBI + OD)	[(GBP5 + DUSP3) / 2] – KLF2	Multicohort analysis (Case- control)	France, Malawi, South Africa, United Kingdom, USA	Adults	Mixed	Genome-wide RNA Microarray	Significance thresholding and forward search
**Walter47 (32) ^39^**	2016	Diagnostic (TB vs OD)	SVM	Case-control	USA	Adults	Negative	Genome-wide RNA Microarray	SVM with recursive feature elimination
**Walter51 (46) ^39^**	2016	Diagnostic (TB vs LTBI)	SVM	Case-control	USA	Adults	Negative	Genome-wide RNA Microarray	SVM with recursive feature elimination
**Walter119 (101) ^39^**	2016	Diagnostic (TB vs LTBI + OD)	SVM	Case-control	USA	Adults	Negative	Genome-wide RNA Microarray	SVM with recursive feature elimination
**Zak16 ^40^**	2016	Prognostic (Incipient TB vs LTBI)	Pair-wise SVM ensemble	Case-control nested in prognostic cohort	South Africa	Adolescents	Negative	Genome-wide RNA Sequencing	SVM-based gene pair approach

* A study may evaluate multiple signatures using several validation cohorts. Studies and cohorts are designated by the first author name and year of publication (e.g. Author2019a) and signatures by first author and number of transcripts (e.g. Author11). Numbers in brackets indicate the subsequently reduced number of transcripts in a given signature due to duplicate transcript symbols (IDs) or where transcript sequences from an original discovery cohorts could not be mapped to a more recent reference transcriptome. LTBI, latent tuberculosis infection. OD, other diseases. HC, healthy controls. SVM, support vector machines. LASSO, Least Absolute Shrinkage and Selection Operator. FS-PLS, Forward Selection-Partial Least Squares. RT-qPCR, real-time quantitative polymerase chain reaction. Dc-RT MLPA, dual-color-Reverse-Transcriptase-Multiplex-Ligation-dependent-Probe-Amplification.

### Transcriptomic signatures included in quantitative synthesis

All 31 signatures evaluated were discovered in case-control cohorts, eight of which were nested within prospective cohorts ( Table 3). Only one of the 31 signatures (Rajan5) ^44^ was discovered in a cohort exclusively consisting of PLHIV. Seven signatures (Duffy10 ^43^, Gliddon3 ^55^, Gliddon4 ^55^, Kaforou27 ^37^, Kaforou44 ^37^, Kaforou53 ^37^, and Sweeney3 ^19, 49^) were discovered using the Kaforou2013 cohort ^37^ and two signatures (Anderson42 and Anderson51) ^38^ using the Anderson2014 cohort ^38^, which included participants both with and without HIV. The 31 signatures were predominantly discovered in adult cohorts, with only 6 out 31 derived in paediatric (Gjøen7 ^51^, Anderson42, and Anderson51) or adolescent (Darboe11 ^56^, Penn-Nicholson6 ^45^, and Zak16 ^40^) cohorts. Signature discovery cohorts were geographically diverse, with recruitment in Asia (3 signatures), Europe (10), North America (4), and South America (1); however there was a predominance in Africa (16), particularly South Africa (15).

The 40 most frequent transcripts, all included in 3 or more signatures, are listed in Table 4. We used the INTERFEROME database ^57^ to classify interferon-stimulated genes (ISGs): We defined ISGs as genes significantly up or down regulated in expression (>1.5 fold change) in any human samples treated with Type-I IFN, relative to control samples. Almost all (38/39) of the most common transcripts with available gene annotations were classified as ISGs. The six transcripts most frequently included in signatures were Guanylate Binding Protein (GBP) 5 (11 signatures), GBP6 (10 signatures), Complement C1q B Chain (C1QB) and Fc fragment of IgG receptor Ia (FCGR1A) (7 signatures each), and Basic Leucine Zipper ATF-Like Transcription Factor 2 (BATF2) and GBP2 (6 signatures each).

**Table 4.** Forty most frequent transcripts included in 3 or more transcriptomic signatures, sorted by transcript use frequency.

Gene ID	Transcript use frequency	*Interferon- stimulated gene	Anderson42 (39)	Anderson51 (39)	Berry393	Berry86	Darboe11	de Araujo1	Duffy10	Gjøen8	Gliddon3	Gliddon4	Huang13 (11)	Kaforou27 (25)	Kaforou44 (39)	Kaforou53 (45)	Maertzdorf4
GBP5	11	Yes	1	1	1		1			1						1	
GBP6	10	Yes	1	1	1				1			1		1	1	1	
C1QB	7	Yes		1	1				1		1		1	1			
FCGR1A	7	Yes			1						1			1		1	
BATF2	6	Yes			1		1										
GBP2	6	Yes			1		1										
ANKRD22	5	Yes			1									1		1	
DUSP3	5	Yes			1									1	1	1	
FCGR1B	5	Yes			1				1					1		1	
GBP1	5	Yes			1		1										1
PRDM1	5	Yes			1							1			1		
SCARF1	5	Yes					1										
SEPT4	5	Yes			1										1	1	
SERPING1	5	Yes			1		1								1		
ALDH1A1	4	**No** ^†^		1	1										1	1	
CYB561	4	Yes		1											1	1	
DHRS9	4	Yes			1	1											
FCGR1C	4	Yes					1							1		1	
GAS6	4	Yes			1									1		1	
IFITM3	4	Yes			1					1							1
LHFPL2	4	Yes			1									1	1	1	
SMARCD3	4	Yes		1	1									1			
STAT1	4	Yes			1		1										
VAMP5	4	Yes		1	1									1			
ACTA2	3	Yes	1		1												
BLK	3	Yes			1											1	
C20ORF103 (LAMP5)	3	Yes		1													
CD74	3	Yes				1									1	1	
CREB5	3	Yes			1										1	1	
DEFA1	3	Yes	1	1												1	
ETV7	3	Yes			1		1										
FER1L3 (MYOF)	3	Yes		1	1	1											
ID3	3	Yes			1				1								1
KREMEN1	3	Yes		1	1												
LOC389386	3	NA **		1											1	1	
OSBPL10	3	Yes		1												1	
POLB	3	Yes				1										1	
TAP1	3	Yes			1		1										
TMCC1	3	Yes										1			1	1	
TRAFD1	3	Yes			1		1										

**Table:** 

Gene ID	Transcript use frequency	*Interferon- stimulated gene	Penn- Nicholson6	Qian17	Rajan5	Roe1	Roe3	Roe4	Roe5	Sambarey10	Singhania20	Suliman2	Suliman4	Sweeney3	Walter47 (32)	Walter51 (46)	Walter119 (101)	Zak16
GBP5	11	Yes					1							1	1		1	1
GBP6	10	Yes			1												1	
C1QB	7	Yes														1		
FCGR1A	7	Yes								1						1		1
BATF2	6	Yes				1	1		1									1
GBP2	6	Yes	1													1	1	1
ANKRD22	5	Yes										1						1
DUSP3	5	Yes												1				
FCGR1B	5	Yes	1															
GBP1	5	Yes		1														1
PRDM1	5	Yes													1		1	
SCARF1	5	Yes					1				1						1	1
SEPT4	5	Yes											1					1
SERPING1	5	Yes	1															1
ALDH1A1	4	**No**†																
CYB561	4	Yes													1			
DHRS9	4	Yes														1	1	
FCGR1C	4	Yes																1
GAS6	4	Yes											1					
IFITM3	4	Yes		1														
LHFPL2	4	Yes																
SMARCD3	4	Yes								1								
STAT1	4	Yes		1														1
VAMP5	4	Yes														1		
ACTA2	3	Yes			1													
BLK	3	Yes											1					
C20ORF103 (LAMP5)	3	Yes														1	1	
CD74	3	Yes																
CREB5	3	Yes																
DEFA1	3	Yes																
ETV7	3	Yes																1
FER1L3 (MYOF)	3	Yes																
ID3	3	Yes																
KREMEN1	3	Yes														1		
LOC389386	3	NA**																
OSBPL10	3	Yes										1						
POLB	3	Yes															1	
TAP1	3	Yes																1
TMCC1	3	Yes																
TRAFD1	3	Yes																1

* Gene significantly up or down regulated in expression (>1.5 fold change) in samples treated with Type-I interferon, relative to control samples, were defined as interferon-stimulated genes (ISGs). Data from INTERFEROME database (Samarajiwa SA, Forster S, Auchettl K, Hertzog PJ. INTERFEROME: the database of interferon regulated genes. *Nucleic Acids Res*. 2009;37:D852-7). ** No updated gene annotation available. NA, not available. ^† ^No experimental data demonstrating >1.5 fold-change in expression in response to Type-I interferon, however, there was >1.5 fold-change in expression response to Type-II interferon.

### Quality appraisal of eligible studies

**Figure 2.**
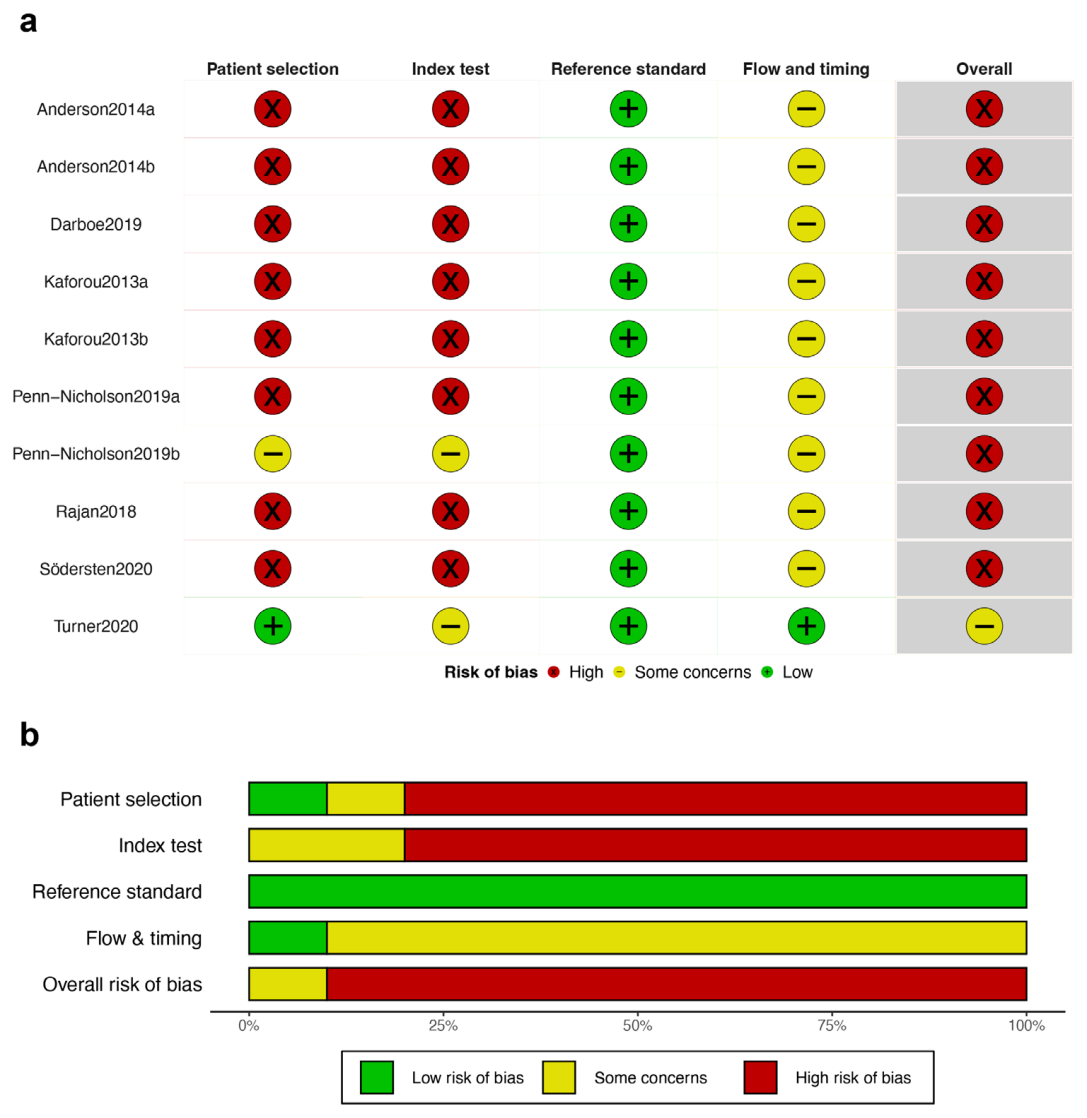
Quality Assessment of Diagnostic Accuracy Studies-2 (QUADAS-2). ( **a**) Assessment of bias in individual cohorts included in the systematic review and ( **b**) summary of results of the QUADAS-2 assessment in the four domains: patient selection, index test, reference standard, and flow and timing.

In the patient selection domain, 5 of the 10 independent test and external validation cohorts consecutively or randomly enrolled participants, with the remaining cohorts not reporting sampling method ( Figure 2). Eight of the 10 cohorts utilised a case-control design ( Table 2), with exclusion of participants with uncertain diagnosis introducing a high risk of spectrum bias and potentially inflating diagnostic accuracy. The Penn-Nicholson2019b cohort ^45^ used a prospective design in recruiting symptomatic clinic attendees, but excluded probable and uncertain TB cases from analysis. Only one prospective diagnostic accuracy study, Turner2020 ^13^, measured signature scores and tested performance in all enrolled participants with clinically suspected tuberculosis, including those with uncertain diagnosis, representative of the target population of symptomatic clinic attendees.

In the index test measurement domain, transcriptomic signature scores were interpreted without knowledge of the reference standard (i.e. blinded) in the Turner2020 ^13^ and Penn-Nicholson2019b ^45^ cohorts, with unclear reporting for the other studies. Due to the early stage of biomarker development and diverse signature measurement and score calculation methodologies, no studies used pre-specified signature score thresholds. The risk of bias in the reference standard domain was deemed to be low in all cohorts with use of appropriate and standardised microbiological confirmatory TB testing (Mtb culture and Xpert MTB/RIF) likely to correctly classify the target condition. Reference standard results were interpreted without knowledge of the results of the transcriptomic signature scores (i.e. blinded) for all included studies. In the study flow and timing domain, all studies used an appropriate interval between index test and reference standard sample collection, and all participants received the same reference standard tests. However, only the Turner2020 ^13^ study included all participants in analysis. In terms of applicability concerns, selection of participants, and measurement and interpretation of index tests and reference standard matched the review question for all included studies. Overall 9 of the 10 cohorts had a high risk of bias, and one study (Turner2020 ^13^) had some concerns due to lack of a pre-specified test threshold prospectively applied to each signature ( Figure 2).

### Transcriptomic signature diagnostic performance

**Figure 3.**
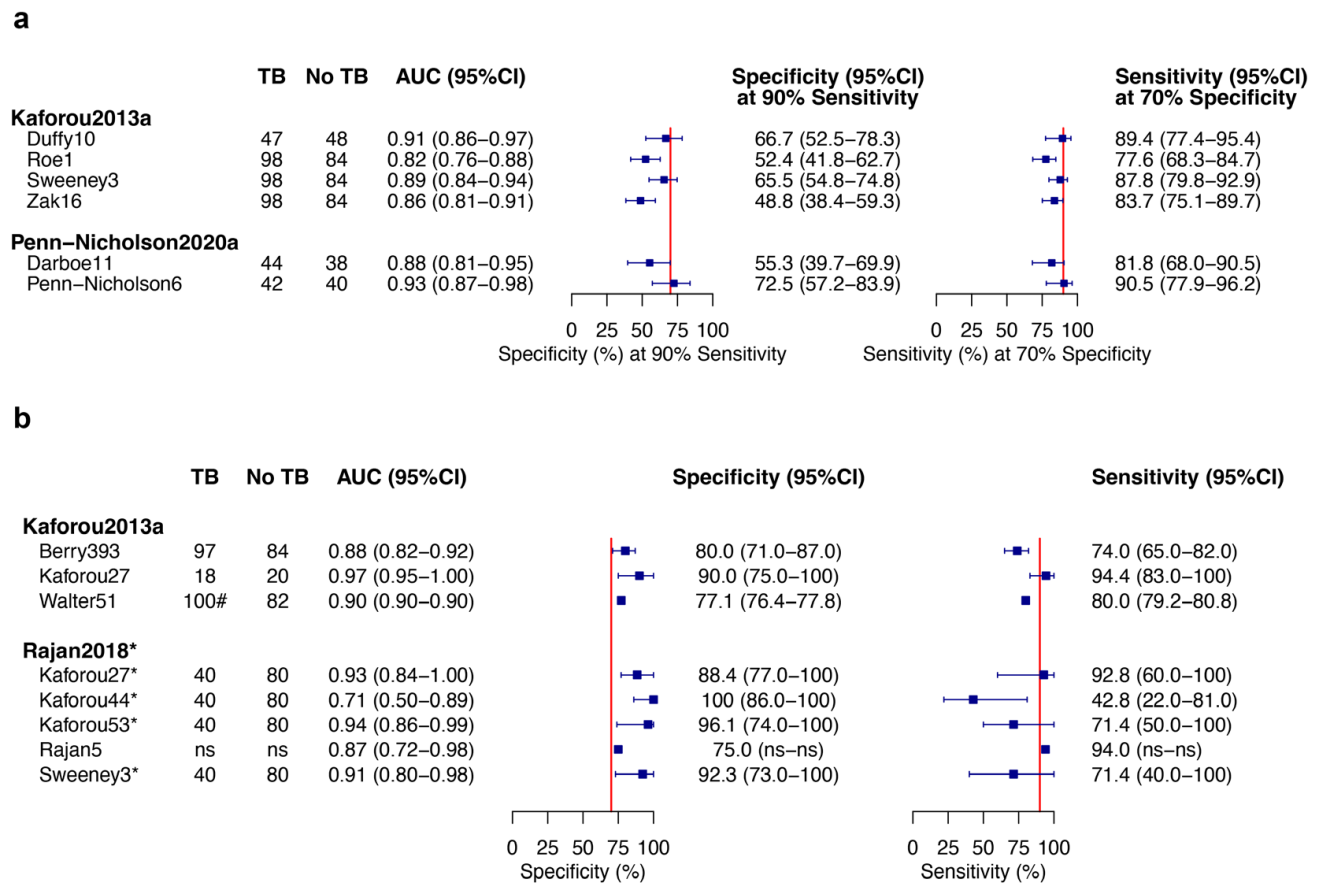
Transcriptomic signature diagnostic performance for differentiation of patients with microbiologically confirmed TB disease from latent-Mtb infected individuals or healthy controls in independent test or external validation cohorts. ( **a**) Signature diagnostic performance (area under the curve, AUC) recalculated using participant-level data retrieved from supplementary data or supplied by study authors, with sensitivity and specificity benchmarked against the WHO TPP minimum performance criteria for a triage test (70% specificity and 90% sensitivity, red vertical lines) ^16^. 95% confidence intervals for AUC and proportions (sensitivity and specificity) were calculated using the DeLong ^30^ and binomial proportion (Wilson) ^31^ methods, respectively. ( **b**) Signature diagnostic performance and 95% confidence intervals as originally reported in studies where participant-level data were not available. *Data for other signatures measured in the full Rajan2018 cohort were extracted from an ATS Conference abstract (Rajan, *AJRCCM* 2017) ^47^. The number of participants included in the Rajan2018 test set was not specified (ns). ^#^100 TB cases were reported in the paper by Walter *et al.* ( *J Clin Microbiol,* 2016) ^39^, however only 97 TB cases were included in the original microarray analysis ^37^.

**Figure 4.**
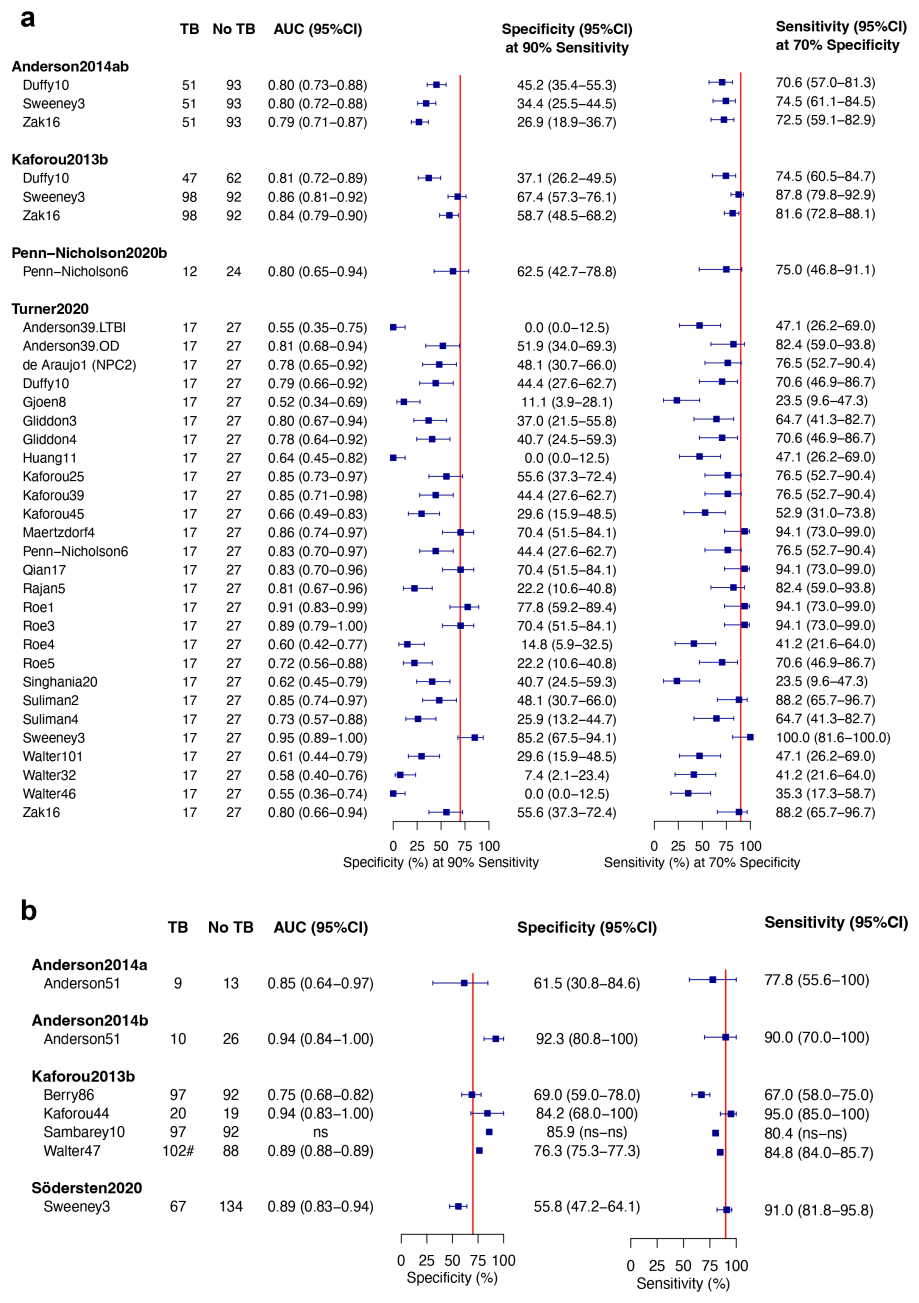
Transcriptomic signature diagnostic performance for differentiation of patients with microbiologically confirmed TB disease from patients with other diseases in independent test or external validation cohorts. ( **a**) Signature diagnostic performance (area under the curve, AUC) recalculated using participant-level data retrieved from supplementary data or supplied by study authors, with sensitivity and specificity benchmarked against the WHO TPP minimum performance criteria for a triage test (70% specificity and 90% sensitivity, red vertical lines) ^16^. 95% confidence intervals for AUC and proportions (sensitivity and specificity) were calculated using the DeLong ^30^ and binomial proportion (Wilson) ^31^ methods, respectively. ( **b**) Signature diagnostic performance and 95% confidence intervals as originally reported in studies where participant-level data were not available. ^#^102 TB cases were reported in the paper by Walter *et al.* ( *J Clin Microbiol,* 2016) ^39^. however only 97 TB cases were included in the original microarray analysis ^37^. ns, not specified.

Nine independent test or external validation diagnostic cohorts with PLHIV subgroups were included in the systematic review ( Table 2). Three cohorts evaluated diagnostic performance of 12 signatures for discriminating HIV-infected adults with prevalent TB disease from latent-Mtb infected individuals or healthy controls with HIV ( Figure 3), and 6 cohorts evaluated diagnostic performance of 29 signatures for discriminating HIV-infected adults or children with prevalent TB disease from those with HIV and other respiratory or systemic diseases ( Figure 4).

Only the Kaforou27 (Kaforou2013a and Rajan2018 cohorts), Penn-Nicholson6 (Penn-Nicholson2020a cohort), and Rajan5 (Rajan2018 cohort) signatures met the WHO TPP minimum performance criteria for a triage test for differentiating adults with prevalent TB disease from latent-Mtb infected individuals or healthy controls with HIV in these case-control studies ( Figure 3). However, the upper bounds of the 95% confidence intervals for 4 other signatures met the TPP benchmarks: Darboe11 (Penn-Nicholson2020a cohort), Duffy10 (Kaforou2013a cohort), Kaforou53 (Rajan2018 cohort), and Sweeney3 (Kaforou2013a and Rajan2018 cohorts).

Signatures which are able to distinguish PLHIV with prevalent TB disease from those with other respiratory or systemic diseases are more clinically useful in the inpatient or outpatient setting, as compared to the community. Seven out of 29 signatures met the WHO TPP minimum performance criteria for a triage test in this context ( Figure 4): Anderson51 in the Anderson2014b paediatric cohort; Kaforou44 in the Kaforou2013b adult cohort; and Maertzdorf4, Qian17, Roe1, Roe3, and Sweeney3 in the Turner2020 adult cohort. A further 8 signatures (Anderson39.OD, de Araujo1, Kaforou25, Kaforou39, Penn-Nicholson6, Rajan5, Suliman2, and Zak16) had upper 95% confidence interval bounds for sensitivity which met the 90% TPP benchmark (with specificity set at 70%) in the Turner2020 prospective diagnostic accuracy cohort. However, the PLHIV subgroup of this cohort only included 17 TB cases and 27 controls with other respiratory diseases. In other cohorts, the Anderson51 (Anderson2014a cohort), Penn-Nicholson6 (Penn-Nicholson2020b cohort), and Sweeney3 (Kaforou2013b cohort) signatures also approached the WHO minimum TPP triage test criteria. Only one signature (Sweeney3) was tested using a point-of-care device, the Cepheid Xpert-MTB-HR Prototype in the Södersten2020 cohort on biobanked whole blood RNA samples from symptomatic inpatients with HIV. Sweeney3 achieved sensitivity of 91.0% (95%CI 81.8–95.8) and specificity of 55.8% (95%CI 47.2–64.1), falling short of the WHO TPP benchmark. No signatures met the WHO TPP benchmark criteria for a non-sputum confirmatory diagnostic test in PLHIV (98% specificity and 80% sensitivity) ^16^ in any cohorts.

**Figure 5.**
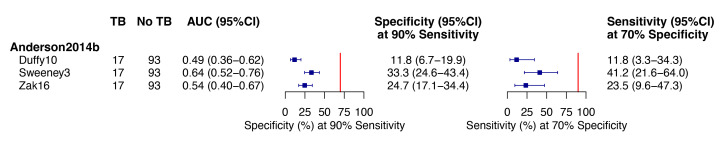
Transcriptomic signature diagnostic performance for non-microbiologically confirmed presumptive TB disease. Transcriptomic signature diagnostic performance for differentiation of children with non-microbiologically confirmed (i.e. presumptive clinically-diagnosed) TB disease from children with other diseases in an external validation cohort. Signature diagnostic performance (area under the curve, AUC) recalculated using participant-level data supplied by study authors, with sensitivity and specificity benchmarked against the WHO TPP minimum performance criteria for a triage test (70% specificity and 90% sensitivity, red vertical lines) ^16^. 95% confidence intervals for AUC and proportions (sensitivity and specificity) were calculated using the DeLong ^30^ and binomial proportion (Wilson) ^31^ methods, respectively.

Participant-level data were available for only 3 signatures in the Anderson2014b cohort, which also included 17 children with non-microbiologically confirmed, presumptive clinically-diagnosed TB disease ( Table 2). All signatures performed poorly in differentiating TB from other diseases in this subset ( Figure 5).

Only the Södersten2020 study reported signature performance stratified by CD4 cell count, with lower Sweeney3 specificity in the 56 inpatients with CD4 cell count less than 200 (66.7%, 95%CI 46.7–82.0) as compared to 129 inpatients with CD4 greater than or equal to 200 (94.7%, 95%CI 88.9–97.5) ^46^. Conversely, sensitivity was higher in the inpatients with CD4 cell count less than 200 (93.8%, 95%CI 79.9–98.3) as compared to the inpatients with CD4 greater than or equal to 200 (75.0%, 95%CI 50.5–89.9). No participant-level data were available to perform further subgroup analyses by CD4 cell count, HIV plasma viral load, TPT or ART status, or other variables in any cohorts.

There was considerable clinical and methodological heterogeneity between cohorts, with participants recruited in diverse settings, with dissimilar eligibility criteria, and distinct composition of the control groups. Studies also used different signature measurement methods (microarray, RNA sequencing, and RT-qPCR), different methods of signature score calculation, and there were no standardised signature score thresholds. Due to the significant heterogeneity in study design and index test measurement, and limited participant data, a meta-analysis was not deemed appropriate.

### Transcriptomic signature prognostic performance

**Figure 6.**
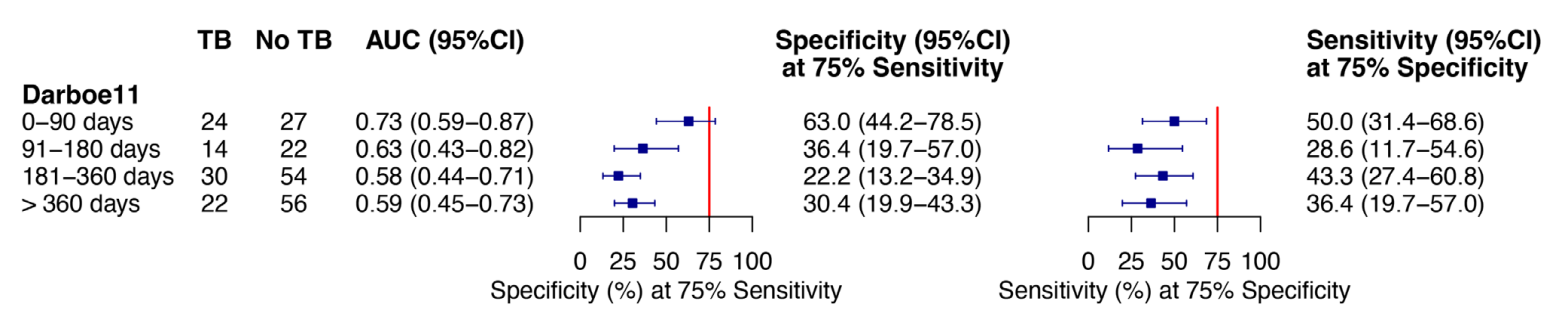
Transcriptomic signature prognostic performance for microbiologically confirmed TB disease. Darboe11 transcriptomic signature prognostic performance for microbiologically confirmed recurrent TB disease in the TB Recurrence upon Treatment with HAART (TRuTH) cohort (Darboe2019) ^42^. Signature prognostic performance (area under the curve, AUC) is stratified by time window following completion of TB treatment: 0–90, 91–180, 181–360, and >360 days. Sensitivity and specificity benchmarked against the WHO TPP minimum performance criteria for a prognostic test (75% sensitivity and 75% specificity, red vertical lines) ^18^.

Only one study (Darboe2019) ^42^ evaluated transcriptomic signature prognostic performance for recurrent TB disease in adults with HIV who had recently completed TB therapy. There were no eligible studies evaluating performance for prediction of progression to incident TB disease in PLHIV without prior TB disease. We stratified the prognostic performance of the Darboe11 signature by time from measurement until TB disease recurrence ( Figure 6). Signature prognostic performance was best within 90 days of signature measurement, and waned thereafter. Sensitivity and specificity of the Darboe11 signature did not meet the minimum WHO TPP performance criteria for a prognostic test (75% sensitivity and 75% specificity) ^18^ in any time window. Sample size was not sufficient to perform subgroup prognostic performance analyses, however Darboe11 signature scores were higher in individuals with detectable plasma HIV viral load (>400 copies/mL) as compared to those with an undetectable plasma viral load (<400 copies/mL; p<0.0001) ^42^.

### GRADE evidence summary

A total of 10 cohorts, 2 prospective cohorts (29 TB cases; 51 other respiratory diseases) and 8 case-control studies (353 TB cases; 606 controls), were included in this systematic review of the diagnostic and prognostic accuracy of host blood transcriptomic signatures in PLHIV. All studies used reliable reference standards for definitive TB diagnosis. However, we adjudged that there was a very serious risk of bias due to exclusion of participants with indeterminate (non-microbiologically confirmed) TB in numerous studies, removing diagnostic uncertainty, and resulting in reduced diversity of clinical TB disease. Several case-control studies also included healthy asymptomatic controls, healthy Mtb-sensitised (latent Mtb infected) individuals (IGRA or TST positive), or individuals with other uncommon diseases, not reflective of the target population or setting, further exacerbating the spectrum bias. The inclusion of severe TB cases and healthy controls (or controls with inappropriate other diseases) may have resulted in misleadingly high diagnostic accuracy in some of these studies. Other limitations included uncertainty regarding consecutive recruitment, blinding status not clearly stated, and lack of *a priori* score thresholds.

Indirectness is synonymous with applicability, generalisability, translatability, and external validity of the evidence ^36^. Included studies evaluated diagnostic performance among adults or children, within clinical outpatient and hospital inpatient settings, prospectively among symptomatic clinic attendees or within matched case-control cohorts. Some of these settings and populations are not appropriate or relevant to clinical practice, and results are unlikely to be generalisable. The lack of diagnostic uncertainty, spectrum bias, and inappropriate control selection is a concern for external validity of these results. Point-of-care device translatability has only been tested for one signature (Sweeney3) among PLHIV, with unsatisfactory diagnostic accuracy ^46^. Technical variability and operator reliability have not been tested on point-of-care platforms for any tests.

With regards to downstream effects, false negative signature results (patients incorrectly classified as not having TB) may have serious consequences, with delayed TB diagnosis resulting in Mtb transmission to close contacts, and increased risk of morbidity and mortality. While consequences are less serious, false positive results (individuals incorrectly classified as having TB) may result in costly further investigations, or 6 months of curative therapy with potential adverse effects and without apparent benefit. Incorrect diagnosis of TB may result in missed or delayed alternate diagnosis and treatment, with potential downstream consequences. Misdiagnosis of TB may also result in stigmatisation from family and community, and psychological distress. There is no uncertainty regarding true positive and true negative results.

We found very serious risks of inconsistency, with significant unexplained heterogeneity of diagnostic sensitivity and specificity estimates for signatures in different validation cohorts and settings. No signatures consistently met the WHO TPP criteria in all or most cohorts, suggesting publication bias toward more optimistic signature performance, particularly in discovery cohorts. There was also a very serious risk of imprecision, with small sample sizes and wide confidence intervals for estimates of test accuracy among PLHIV, with no pooling of data. Data was not available or accessible for the PLHIV subgroup in numerous cohorts. In summary, the data included in this review provides very low quality evidence and we would not recommend any changes to clinical practice based on these results.

## Discussion

TB transcriptomic biomarkers selected for advancement through the diagnostics pipeline for further development as point-of-care tests should ideally perform well in PLHIV. We systematically searched online databases for studies which evaluated the performance of host-blood transcriptomic signatures for diagnosing prevalent TB and identifying those who are progressing to incident TB in PLHIV, and compared performance to the WHO TPP criteria. We found 12 studies published prior to 2021 which included 10 independent test or validation cohorts featuring PLHIV, evaluating 31 transcriptomic signatures. Several of the signatures approached or met the WHO TPP minimum performance criteria for a triage test for differentiating people with prevalent TB disease from latent-Mtb infected individuals, healthy controls, or individuals with other respiratory or systemic diseases ^16^. However, no transcriptomic signatures met the TPP benchmark criteria for a non-sputum confirmatory diagnostic test among PLHIV. The signatures also performed poorly for diagnosing non-microbiologically confirmed, presumptive TB disease.

Only one cohort evaluated a signature for predicting TB disease recurrence in individuals who recently completed TB treatment and initiated ART. Prognostic performance appeared to be superior proximally to incident TB disease, with highest AUC and specificity in the 3 months preceding TB recurrence ^42^. The Darboe11 signature did not meet the WHO TPP for a prognostic test in any time window in this population ^18^.

Among the 31 signatures evaluated, we found that the genes most frequently incorporated in TB transcriptomic signatures were ISGs, which may also be upregulated by chronic HIV viraemia ^62, 63^. We hypothesised that these signatures would be less discriminatory for TB in PLHIV, particularly among viraemic ART-naïve individuals, due to an increased abundance of circulating type-I IFNs. While no studies performed subgroup analyses of diagnostic accuracy by HIV plasma viral load, Darboe and colleagues ^42^ reported higher signature scores in individuals with a viral load greater than 400 copies per mL, as compared to those with an undetectable viral load (<400 copies/mL). Södersten and colleagues ^46^ demonstrated decreased Sweeney3 specificity in adults with a CD4 cell count less than 200, as compared to those with CD4 cell count greater than 200. Low CD4 cell count is a proxy for ART-naivety and high HIV plasma viral load. The lower specificity is possibly due to higher signature scores in the control group, either due to HIV viraemia, undiagnosed early or minimal TB, or other opportunistic infections. By specifically excluding ISGs, Esmail and colleagues ^62^ have demonstrated that classical complement pathway and Fc-γ receptor 1 (FCGR1) genes are also differentially expressed in individuals with subclinical HIV-associated TB. While traditional discriminant analysis yields an overabundance of ISGs and an underabundance of B- and T-cell genes in active TB patients versus latently Mtb-infected controls, Singhania and colleagues ^53^ have shown that a modular approach (i.e. pre-filtering genes by functional modules) results in a more diverse gene set.

The synthesised systematic review results represent an overall low quality of evidence, with lack of generalisability and external validity, inconsistency in results between studies, and imprecision in estimates. Most of the discovery and validation studies were conducted in Africa, particularly South Africa, limiting geographic diversity and generalisability of results. There were also few training and test datasets including PLHIV, with a notable overreliance on the Kaforou2013 and Anderson2014 datasets for signature discovery and validation, further limiting generalisability. Also, all eligible validation cohorts were from outpatient or inpatient settings. It is notable that signatures generally performed best in small test sets derived from the same population as the signature training cohort (e.g. Rajan5 in Rajan2018 test cohort, Kaforou27 in Kaforou2013a test cohort, Kaforou44 in Kaforou2013b, Anderson51 in Anderson2014b, and Penn-Nicholson6 in Penn-Nicholson2020a cohort), and performance waned in subsequent external validation. The differences in signature performance between cohorts, with signatures meeting WHO TPP criteria in one cohort but not others, is also likely attributable to differences in discovery and validation cohort designs, and publication bias. Multicohort gene meta-analytical methods, similar to those employed by Sweeney and colleagues ^49^, may help to overcome such limitations, and result in greater reproducibility of performance across cohorts.

There is insufficient evidence in the literature to suggest that signatures discovered in cohorts of PLHIV only (e.g. Rajan5) have greater diagnostic accuracy in populations with HIV than signatures discovered among HIV-uninfected (e.g. Darboe11, Penn-Nicholson6, Roe1, and Roe3) or mixed (e.g. Duffy10, Kaforou27, and Sweeney3) cohorts. However, there is a need for larger prospective cohorts, which include PLHIV, for signature discovery and validation, to confirm the utility of transcriptomic signatures among PLHIV. Such cohorts should enrol clearly defined, clinically relevant populations, such as symptomatic clinic attendees, HIV-infected outpatients and other high risk groups; or healthy, asymptomatic individuals from communities in diverse high incidence settings. To reduce spectrum bias, all participants should be enrolled and included in analysis, irrespective of diagnostic uncertainty. Prolonged follow-up over 3 to 6 months may aid diagnosis in unconfirmed cases. Most studies used microarray and RNA sequencing to measure signature scores. Only a few signatures were tested using near-point-of-care benchtop PCR instruments, and one with a point-of-care RT-qPCR device ^46^. Future field evaluation studies should move towards technologies implementable at the point-of-care, such as the Cepheid Xpert-MTB-HR Prototype ^46, 64, 65^.

Strengths of this systematic review include the comprehensive search strategy, with rigorous eligibility criteria, and publication of a peer-reviewed study protocol. The review also had several limitations. The pre-specified literature search strategy only included studies published prior to 2021; there were few studies with data available for an PLHIV subgroup in test or validation sets in this period, and most included cohorts were small and underpowered. The use of published summary performance data is also problematic due to different statistical methods used in original papers. We were also unable to obtain summary diagnostic performance estimates, or participant-level data to reconstruct two-by-two tables, for PLHIV subgroups from 9 studies, despite contacting corresponding authors. Most papers did not conform to the standard reporting guidelines for diagnostic accuracy studies (STARD) ^66^, with missing information particularly relating to study design, participant recruitment, and blinding to reference standard result. Requisite anonymised participant data with signature scores were only available for a handful of studies. The lack of participant-level metadata precluded subgroup analyses. We were thus unable to systematically determine the effect of HIV viral load, CD4 cell count, TPT, and ART on transcriptomic signature diagnostic performance. Metanalysis was also deemed inappropriate due to the clinical and methodological heterogeneity and high risk of bias in cohort design and signature measurement method, with no predetermined score cut-offs or methods of standardising scores across platforms.

In the two years subsequent to the completion of this systematic literature review, diagnostic and prognostic performance of several transcriptomic signatures measured by qPCR were prospectively tested for mass screening in a South African community setting amongst predominantly asymptomatic PLHIV who were not seeking care ^21, 67^. Most transcriptomic signatures measured in this cohort met WHO triage test TPP benchmark criteria among symptomatic participants, however the signatures were upregulated by respiratory viral infection and HIV viraemia, and offered poor specificity for diagnosing sub-clinical TB. The Roe1 signature met the WHO prognostic test TPP benchmarks through 15 months of follow-up among PLHIV, and Darboe11 and Roe3 approached this threshold. These signatures could enable the screening of symptomatic adults seeking care and predict risk of progression to TB disease, thus enabling targeting of preventive therapy ^68^.

Sputum-free TB diagnostics remain an appealing prospect in populations who are unable to produce sputum, such as children and non-ambulant hospitalised patients, and among individuals with paucibacillary or negative sputum samples, such as disseminated TB disease cases or those with advanced HIV ^69– 71^. Recent studies have evaluated transcriptomic signature diagnostic performance for extrapulmonary TB and TB immune reconstitution inflammatory syndrome (IRIS), both of which are difficult to diagnose, and have high morbidity and mortality among PLHIV. The Sweeney3 and the Penn-Nicholson6 signatures were shown to predict TB immune reconstitution inflammatory syndrome (IRIS) prior to ART initiation and at IRIS diagnosis in children, and paradoxical worsening of TB within half a week of starting ART in adults with TB disease ^72^. The signatures were also able to distinguish IRIS in adults with TB meningitis from HIV-infected patients who did not develop IRIS after starting ART. In this context, transcriptomic signatures could potentially be used to delay ART initiation to prevent paradoxical worsening of TB. While performance is promising, these studies are generally underpowered with clinically inappropriate control groups, suffering from similar flaws in study design as discussed previously. Larger, prospective studies are required to validate transcriptomic signatures for diagnosis of disseminated TB and TB IRIS.

Additionally, most of the cohorts evaluated in this systematic review included adults, with only the Anderson2014 paediatric cohort eligible for inclusion. Paediatric TB is particularly difficult to diagnose due to its paucibacillary nature and difficulty in obtaining sputum samples from small children ^73– 76^. An accurate non-sputum diagnostic, such as host response transcriptomic signatures, would transform the diagnosis of TB in children ^77^. However, young children are frequent vectors for respiratory and gastrointestinal viruses, and specificity of transcriptomic signatures may be low due to induction of ISG signalling from viral infections.

Transcriptomic signatures show promise for screening for prevalent TB to guide further investigations and predicting progression to incident TB for targeted TB preventive therapy ^17^. However, evidence among PLHIV is limited and mostly from small case-control studies with high risk of spectrum bias. This review emphasises the need for larger heterogenous prospective discovery and validation cohorts exclusively consisting of PLHIV, or mixed cohorts which include PLHIV. Such cohorts should ideally comparatively test biomarker performance side-by-side to determine which signature should be advanced through the developmental pipeline. Further research exploring the effect of HIV clinical, virological, and immunological status is necessary for the design and implementation of TB transcriptomic signatures in this population who are at heightened risk of TB and its sequelae. 

## Data Availability

All data underlying the results are available in the original published manuscripts or on request from corresponding authors. No original data are associated with this article.

## Reporting guidelines

The PRISMA flow diagram and reporting checklist have been deposited in Zivahub ( https://doi.org/10.25375/uct.21907296), an open access data repository hosted by the University of Cape Town’s institutional data repository powered by Figshare for Institutions.
