# Supplementary material for: Systematic review of diagnostic and prognostic host blood transcriptomic signatures of tuberculosis disease in people living with HIV
Source: Gates Open Res. 2023 May 5;7:27. Originally published 2023 Feb 3. [Version 2] doi: 10.12688/gatesopenres.14327.2 (PMC10133453; doi:10.12688/gatesopenres.14327.2)
Supplement: Peer review history

# Peer review: Reviewer response for version 2

**DOI:** 10.21956/gatesopenres.16010.r33155

**Published:** 2023-05-09

**Authors:** Theresa Ryckman

**Affiliations:** 1 Bloomberg School of Public Health, Department of Epidemiology, Johns Hopkins University, Baltimore, Maryland, USA

I approve of the manuscript and have no further comments.

Are the rationale for, and objectives of, the Systematic Review clearly stated?

Yes

Is the statistical analysis and its interpretation appropriate?

Yes

Are sufficient details of the methods and analysis provided to allow replication by others?

Yes

Are the conclusions drawn adequately supported by the results presented in the review?

Yes

Reviewer Expertise:

Infectious disease epidemiology and policy

I confirm that I have read this submission and believe that I have an appropriate level of expertise to confirm that it is of an acceptable scientific standard.

# Peer review: Reviewer response for version 2

**DOI:** 10.21956/gatesopenres.16010.r33154

**Published:** 2023-05-09

**Authors:** Nagasuma Chandra

**Affiliations:** 1 Department of Biochemistry and Center for BioSystems Science and Engineering (BSSE), Indian Institute of Science, Bengaluru, Karnataka, India

No further comments

Are the rationale for, and objectives of, the Systematic Review clearly stated?

Yes

Is the statistical analysis and its interpretation appropriate?

Yes

Are sufficient details of the methods and analysis provided to allow replication by others?

Yes

Are the conclusions drawn adequately supported by the results presented in the review?

Yes

Reviewer Expertise:

Systems Biology, Tuberculosis, Genomics and Bioinformatics, Diagnostic biomarkers

I confirm that I have read this submission and believe that I have an appropriate level of expertise to confirm that it is of an acceptable scientific standard.

# Peer review: Reviewer response for version 1

**DOI:** 10.21956/gatesopenres.15638.r32952

**Published:** 2023-04-26

**Authors:** Nagasuma Chandra

**Affiliations:** 1 Department of Biochemistry and Center for BioSystems Science and Engineering (BSSE), Indian Institute of Science, Bengaluru, Karnataka, India

The authors report a review of diagnostic and prognostic host-blood transcriptomic signatures of TB in HIV background. They study 31 transcriptomic signatures collated from multiple studies reported for differentiating samples of HIV with HIV co-infections with active or latent TB as well as with other diseases.

 It is indeed a very important topic as there is a great need for accurate diagnosis of TB in HIV background, so that appropriate treatment can be provided, and the spread of TB can be checked. The need for the analysis is well brought out in the review.

 The authors have conducted a systematic survey for relevant transcriptomic data reported over a period of 30 years and use well-defined criteria for inclusion and exclusion of the signatures, and list clear objectives and evaluation metrics and also quality assessment of the diagnostic accuracy. The manuscript is overall well written. It would be helpful if the authors could elaborate on the following points, so as to make it more accessible to a wider audience.

1. A brief note could be added on the WHO TPP minimum performance criteria, and why some datasets did not qualify - especially to bring in the difference between 'lack of required data/comparisons' in the original study versus 'signatures not performing well enough'.
2. A brief introduction on the risk of bias and the various domains under which it is assessed and how it is addressed. 
3. The statement " *Overall 9 of the 10 cohorts had a high risk of bias, and one study (Turner2020) had some concerns*" could be followed by some recommendation on how to read meaning from these studies. It would also help if the term 'concerns' were explained and placed in the risk spectrum.
4. As some studies include more than one sub-objective of diagnosis/prognosis, it would help if a note is added in discussion on the diagnostic accuracy/potential for each comparison axis separately.

Are the rationale for, and objectives of, the Systematic Review clearly stated?

Yes

Is the statistical analysis and its interpretation appropriate?

Yes

Are sufficient details of the methods and analysis provided to allow replication by others?

Yes

Are the conclusions drawn adequately supported by the results presented in the review?

Yes

Reviewer Expertise:

Systems Biology, Tuberculosis, Genomics and Bioinformatics, Diagnostic biomarkers

I confirm that I have read this submission and believe that I have an appropriate level of expertise to confirm that it is of an acceptable scientific standard.

# Response

**Published:** 2023-04-29

**Authors:** Simon Mendelsohn

Thank you for your thoughtful review of our manuscript. We appreciate your positive feedback and suggestions for improvement. Please find our response to each of your points below:

 1. We include the following note on the WHO TPP minimum performance criteria in the introduction: “… the minimum World Health Organization (WHO) Target Product Profile (TPP) TB triage test performance criteria (sensitivity 90% and specificity 70%) for diagnosing prevalent TB” and “…minimum prognostic benchmarks (sensitivity 75% and specificity 75%)”.

 Regarding  why “some datasets did not qualify - especially to bring in the difference between 'lack of required data/comparisons'”: per published systematic review protocol eligibility criteria (see Mendelsohn, BMJ Open, 2021), we excluded datasets which did not include PLHIV, or where it was not possible to stratify results by HIV status, where only signature discovery performance was reported (no independent validation), or where the controls or cases were not adequately defined. We also excluded studies where we were not able to retrieve participant level data, or calculated signature scores.

 We did not exclude datasets on the basis of “signatures not performing well enough”.

 2. We appreciate your suggestion to include a brief introduction on the risk of bias and how it is assessed. We will revise the “Risk of bias, applicability, and quality of evidence” Methods section to provide clarity.

 3. As per the point above, we will revise the “Risk of bias, applicability, and quality of evidence” Methods section to provide clarity. We will also add clarity as to why the Turner study had some concerns: “due to lack of a pre-specified test threshold prospectively applied to each signature”.  

 4. We have considered diagnostic (paragraph 1) and prognostic (paragraph 2) performance of the signatures separately in the Discussion.

 Thank you again for your valuable feedback. We will incorporate your suggestions into the revised manuscript to improve the clarity and accessibility of our findings.

# Peer review: Reviewer response for version 1

**DOI:** 10.21956/gatesopenres.15638.r32915

**Published:** 2023-03-02

**Authors:** Theresa Ryckman

**Affiliations:** 1 Bloomberg School of Public Health, Department of Epidemiology, Johns Hopkins University, Baltimore, Maryland, USA

This study systematically reviewed publications that assessed the performance of host blood transcriptomic signatures in diagnosing active TB and/or predicting risk of progression to active TB among PLHIV. The study found that most publications were at high risk of bias, and that few signatures met the WHO criteria for a triage test for diagnosis (and none met the criteria for a prognostic test), with variability in signature performance across different cohorts of PLHIV. The study is very clearly written, and in particular the methods were well-described and easy to follow. I have only a few minor suggestions for the authors.

- In the methods, it would be useful to clarify how the risk of bias across each of the 4 domains was translated into the “overall risk of bias” for each study as in Figure 2 (as I don’t believe a method for arriving at this summary measure of bias is clearly defined in QUADAS-2?)
- Methods: In the quality appraisal results/figure 2, it seems as though exclusion of some patients from analysis was evaluated in both the “patient selection” and “study flow and timing” domains. It would be good to clarify to what extent there was overlap across the considerations included in these 2 domains (e.g., if a patient was excluded due to diagnostic uncertainty, was this exclusion assessed only under “patient selection”, or also under “study flow and timing”?), and to justify this overlap (if there was any).
- For signatures that were evaluated across multiple cohorts of the same type (for example, Sweeney3 was evaluated in 3 cohorts in Figure 4a), given that the authors have deemed a meta-analysis not appropriate, it could be useful to expand on how the signatures may have met WHO TPP criteria under 1 cohort but not others. This is described only briefly in the last paragraph of the results. Were there any signatures tested in multiple cohorts that met the criteria in all or most cohorts? If there were any, it seems useful to call these out (despite the acknowledged risks of bias). If there weren’t any, then this also seems to be an important finding (e.g., even with all the risks of bias - that mostly seem to bias studies in the direction of more optimistic performance - there weren’t any tests that consistently met the criteria).
- In the last paragraph – large heterogeneous prospective discovery and validation cohorts that include PLHIV seem like the obvious gold standard. But such studies are also expensive. Do you think there is a case to be made for discovery and initial validation using smaller cohorts/existing microarray data, and then only proceeding to larger studies if these smaller studies show promise? Given the poor performance of many of the signatures, even in the smaller studies reviewed in this paper (if again, the authors generally deem these smaller studies to result on overly optimistic results), it could probably be argued that in most cases, initial validation of a signature in a large prospective cohort will be a waste of money, because that signature is unlikely to perform well. I just wonder if the findings from this study can point the research community in a more strategic direction while still arguing that evaluation in larger prospective cohorts is probably needed before any signature should be adopted into clinical practice.

Are the rationale for, and objectives of, the Systematic Review clearly stated?

Yes

Is the statistical analysis and its interpretation appropriate?

Yes

Are sufficient details of the methods and analysis provided to allow replication by others?

Yes

Are the conclusions drawn adequately supported by the results presented in the review?

Yes

Reviewer Expertise:

Infectious disease epidemiology and policy

I confirm that I have read this submission and believe that I have an appropriate level of expertise to confirm that it is of an acceptable scientific standard.

# Response

**Published:** 2023-04-29

**Authors:** Simon Mendelsohn

Thank you very much for taking the time to carefully review our study and for your constructive feedback. We appreciate your positive comments regarding the clarity of our writing and the description of our methods.

 1. Regarding your first suggestion, we agree that we could have been clearer about how we arrived at the overall risk of bias for each study in Figure 2. We will revise the “Risk of bias, applicability, and quality of evidence” Methods section to provide clarity.

 2. Regarding your second comment, we tried to avoid overlap between the “Patient selection” and “Study flow and timing” domains in QUADAS-2. For the “Patient selection” domain, we specifically asked “Did the study avoid inappropriate exclusions?” per the eligibility criteria (i.e. were the eligibility criteria appropriate for the study design). For example, did the study inappropriately exclude individuals with comorbidities, such as diabetes, or individuals who use drugs.

 For the “Study flow and timing” domain, we asked whether “…all [eligible] patients [were] included in the analysis?” (i.e. was there inappropriate exclusion of eligible/enrolled participants from analysis due to, for example, inconclusive diagnostic test results/diagnostic uncertainty or loss to follow up). However, we acknowledge that there is some subjectivity in this process.

 3. Regarding your third suggestion, we appreciate your suggestion to expand on the performance of signatures across multiple cohorts. We did not identify any signatures that consistently met the WHO TPP criteria in all or most cohorts. We agree that this may suggest bias toward more optimistic performance. It is notable that signatures generally performed best in small test sets derived from the same population as the signature training cohort (e.g. Rajan5 in Rajan2018 test cohort, Kaforou27 in Kaforou2013a test cohort, Kaforou44 in Kaforou2013b, Anderson51 in Anderson2014b, and Penn-Nicholson6 in Penn-Nicholson2020a cohort), and performance waned in subsequent external validation. The differences in signature performance between cohorts, with signatures meeting WHO TPP criteria in one cohort but not others, is also likely attributable to differences in discovery and validation cohort designs. Multicohort gene meta-analytical discovery and validation methods, similar to those employed by Sweeney and colleagues, may help to overcome such limitations.

 We will expand on the above in the Results and Discussion sections.

 4. Regarding your final suggestion, we agree that large, heterogeneous prospective cohorts that include PLHIV are the gold standard for discovering and validating host blood transcriptomic signatures for TB diagnosis and risk prediction. We also acknowledge that such studies are expensive. This review suggests that transcriptomic/gene signatures discovered in smaller studies are unlikely to be reproducible across multiple distinct cohorts. We agree that using multicohort gene meta-analytical methods in existing microarray or sequencing data is a more pragmatic use of resources, and may result in greater reproducibility of performance across new validation cohorts. However, we argue that there is insufficient existing microarray or sequencing data among people living with HIV, hence the need for large prospective cohorts in this population.

 Thank you again for your thoughtful review and helpful suggestions. We will address these points in our revised manuscript.
